# Impact of pretreatment PET/MRI on radiooncologic target delineation in Hodgkin’s lymphoma: a case series

**DOI:** 10.1007/s00066-025-02446-4

**Published:** 2025-08-14

**Authors:** Kai Kröger, Sebastian Lohmann, Michael Oertel, Peter Borchmann, Andrea Kerkhoff, Georg Lenz, Lars Stegger, Hans Theodor Eich

**Affiliations:** 1 Department of Radiation Oncology, University Hospital Muenster, Muenster, Germany; 2 Department I of Internal Medicine, University Hospital of Cologne, Cologne, Germany; 3 Department of Medicine A, Hematology, Oncology and Pneumology, University Hospital Muenster, Muenster, Germany; 4 Department of Nuclear Medicine, University Hospital Muenster, Muenster, Germany

**Keywords:** Hodgkin’s lymphoma, PET/MRI, PET/CT, ISRT, Contouring

## Abstract

**Purpose:**

Positron-emission tomography combined with computed tomography (PET/CT) is the diagnostic standard for patients with Hodgkin’s lymphoma. Positron-emission tomography combined with magnetic resonance imaging (PET/MRI) is an alternative diagnostic modality that reduces radiation exposure to the patient. This study aims to evaluate the potential merits of PET/MRI compared to PET/CT for target delineation for radiotherapy of Hodgkin’s lymphoma.

**Methods:**

Five patients with newly diagnosed Hodgkin’s lymphoma underwent PET/CT imaging directly followed by PET/MRI imaging as part of initial staging. Both modalities were subsequently compared regarding each patient’s diagnosed involved nodal regions. Three of these patients received radiotherapy after the completion of chemotherapy. In the radiotherapy planning CT, different gross tumor volumes and clinical target volumes were contoured for both PET/CT and PET/MRI and quantitatively compared using the Dice coefficient.

**Results:**

No differences regarding the diagnosed disease stage were observed. The delineated tumor and target volumes showed minor differences without clinical significance.

**Conclusion:**

Positron-emission tomography/MRI is a viable option to assure adequate staging and later target delineation in patients with Hodgkin’s lymphoma. Due to the reduction of radiation exposure compared to PET/CT, it might be the preferable option if readily available.

## Introduction

Hodgkin’s lymphoma (HL) is a hematopoietic malignancy characterized by cancerous Reed–Sternberg cells. Combination chemo- and radiation therapy have led to excellent cure rates for decades [1]. Recently, the emergence of innovative antibody-based immunotherapies with favorable safety and efficacy profiles, including the anti-CD30 antibody–drug conjugate brentuximab vedotin and immune checkpoint inhibitors such as pembrolizumab and nivolumab, have significantly improved the treatment paradigm [4, 31]. Given the frequent young age of patients at diagnosis and the high survival rates, in HL treatment, increased attention has been focused on maintaining the efficacy while decreasing the intensity and long-term toxicities of treatment.

As a consequence, the current radiation therapy planning approach is mainly focused on reducing either the dose or the treatment volume.

Treatment volumes have been reduced in size from extended-field (EFRT) via involved-field (IFRT) to involved-site (ISRT) or involved-node radiotherapy (INRT) [10, 11, 22]. Current consensus guidelines advise the sole treatment of initially involved lymph nodes [19]. Consequently, only lymph nodes showing clear signs of malignancy at pretreatment staging, i.e., metabolically and/or morphologically suspected malignancy in diagnostic imaging or histopathologically proven malignancy, are subject to irradiation [26]. The use of fluorodeoxyglucose (FDG) positron-emission tomography combined with computed tomography (PET/CT) has become an essential part of the management of patients with HL.

Positron-emission tomography/CT is the benchmark for staging of various malignant diseases. Particularly in the field of malignant lymphomas, especially Hodgkin’s lymphoma, an enormous evidence base has been created for both initial staging and the assessment of treatment response as well as possible treatment de-escalation [2, 5, 7, 16, 18, 20, 21, 30, 32].

Reduction of the treatment volume compared to historical large-field irradiation is based on both improved radiotherapeutic accuracy as well as improved staging.

To enable precise target delineation in radiotherapy, FDG-PET/CT scans are critical—the better the prechemotherapy imaging the smaller the RT volume [1, 8, 13]. In addition, the availability of improved radiotherapy equipment has enabled more precise volume definition. Modern linear accelerators allow for application of intensity-modulated radiotherapy (IMRT), while on-board imaging systems enable image-guided radiotherapy (IGRT). Advances in artificial intelligence (AI) and medical imaging have further contributed to the feasibility of adaptive radiotherapy: based on daily image guidance, adaptive radiotherapy directly adjusts the treatment plan for anatomical changes, such as weight loss or tumor response [9].

Treatment of HL usually starts with chemotherapy, followed by RT in selected cases. Due to tumor shrinkage after chemotherapy, the correct identification of involved nodal regions in the planning CT can be difficult. To facilitate target definition, a prechemotherapy PET/CT is of importance. Ideally, PET/CT should be performed in the radiotherapy position.

The reliance on images accurately obtained before the start of chemotherapy poses a challenge for radiation oncologists, as they may not always be involved in HL case management from the beginning. Thus, close interdisciplinary exchange within oncology centers is essential for optimal management of the disease.

Positron-emission tomography combined with magnetic resonance imaging (MRI) in one device (PET/MRI) constitutes a modern alternative to PET/CT that combines the depiction of processes on a molecular level by PET and of morphology by MRI. This approach has possible merits over PET/CT, e.g., the better soft tissue delineation of MRI and the reduced radiation exposure due to omitting the whole-body CT scan [12, 15, 17, 24, 27].

This work aims to evaluate the possible merits and weaknesses of PET/MRI compared to PET-CT staging regarding the influence on RT planning in patients with HL.

## Materials and methods

We searched our database for patients with newly diagnosed Hodgkin’s lymphoma who had undergone diagnostic FDG-PET/CT in the predicted RT position directly followed by diagnostic FDG-PET/MRI to avoid additional radiation exposure due to multiple radioactive tracer applications. Five patients fulfilled the criteria and were analyzed. After securing the histopathological diagnosis, staging PET/CT was performed for all five patients. After obtaining informed consent from the patients, the PET/CT protocol was changed from the diagnostic standard positioning to radiation oncology (RO)-specific positioning. While the standard diagnostic PET/CT procedure involves positioning the patient’s arms in an elevated position, the goal of the RO positioning is to achieve the best possible reproduction of the future treatment position during RT. To determine the ideal position for PET/CT, the available imaging data were evaluated to predict the future treatment position. Since all enrolled patients had HL involving the neck, all patients were positioned supine, with the chin elevated and the arms lowered but slightly abducted. Acquisition of PET was started 60 min after injection of 3 MBq per kg bodyweight [18F]FDG, and PET data were acquired for 2 min per bed position (or equivalent when using continuous bed motion). The data were subsequently reconstructed into two image datasets with and without attenuation correction using the manufacturer-supplied iterative reconstruction algorithm (matrix size 200 × 200, pixel size 4.1 × 4.1 mm, slice thickness 3.0 mm). Either diagnostic CT acquired as part of the study or, if not available for the body parts covered by PET, a dedicated native low-dose CT was used for attenuation correction. Additionally, a contrast-enhanced CT scan was acquired (matrix size 512 × 512, pixel size 1.0 mm, slice thickness 3.0 mm, slice spacing 2.0 mm; Ultravist®, Bayer Vital, Germany).

After performing the PET/CT scan, patients were immediately transferred to PET/MRI. Due to the narrow gantry and the required body coil, the patient’s arms were lowered, but no further changes in the patient’s position with respect to the later RT position were possible. The PET data were acquired for 2 min per bed position and subsequently reconstructed into two image datasets, with and without attenuation correction, using the manufacturer-supplied iterative reconstruction algorithm (matrix size 172 × 172, pixel size 4.2 × 4.2 mm, slice thickness 2.0 mm); attenuation correction was based on a standard coronal two-point Dixon sequence and insertion of typical attenuation coefficients according to tissue class. Native T1- and T2-weighted MRI images were acquired concurrently with PET (matrix size 320 × 260, pixel size 1.3 × 1.3 mm, slice thickness 3.0 mm). In addition, contrast-enhanced T1-weighted MRI with fat suppression was acquired separately (Gadovist®, Bayer Vital GmbH, Germany; same image parameters). The PET/CT scan was acquired with a Biograph™ mCT scanner and the PET/MRI scan using a Biograph™ mMR scanner, both manufactured by Siemens Healthineers, Germany.

The diagnostic information of both scans was used for staging. After completing the diagnostic workup, chemotherapy was initiated. Three patients required postchemotherapy RT according to the risk group classification system of the German Hodgkin Study Group (GHSG). The diagnostic workup of the remaining two patients revealed an advanced stage and treatment was therefore based solely on chemotherapy. The diagnostic data of both scans were compared regarding differences in the resulting clinical staging (Table 1).

**Table 1:** Patient characteristics and volume metrics

Patient	Stage before PET	Stage after PET	PET/CT GHSG regions (*n*)	PET/MRI GHSG regions (*n*)	PET/CT-based CTV (cm^3^)	PET/MRI-based CTV (cm^3^)	Difference CTV-PET/MRI vs. CTV-PET/CT (%)	Dice coefficient
1	IIIB	IVB	14 + bone	14 + bone	–	–	–	–
2	IIA	IIA	5	5	135.5	129.8	−4.2	0.89
3	IIA	IIA	5	5	406	428	+5.4	0.86
4	IIB	IIB	5	5	595	596	+0.2	0.88
5	IIB	IVB	13 + bone marrow + spleen	13 + bone marrow + spleen	–	–	–	–

After completion of chemotherapy, the patients needing RT underwent CT for RT planning in a defined position based on the initial PET/CT and PET/MRI scans. This native RT-planning CT was acquired with a slice thickness of 3 mm in free breathing without additional motion management.

**Fig. 1:**
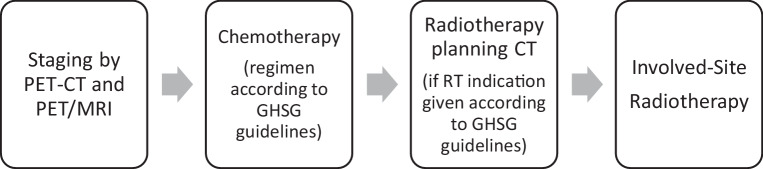
Flowchart of performed scans and therapeutic interventions. *GHSG* German Hodgkin Study Group, *RT* radiotherapy

**Fig. 2:**
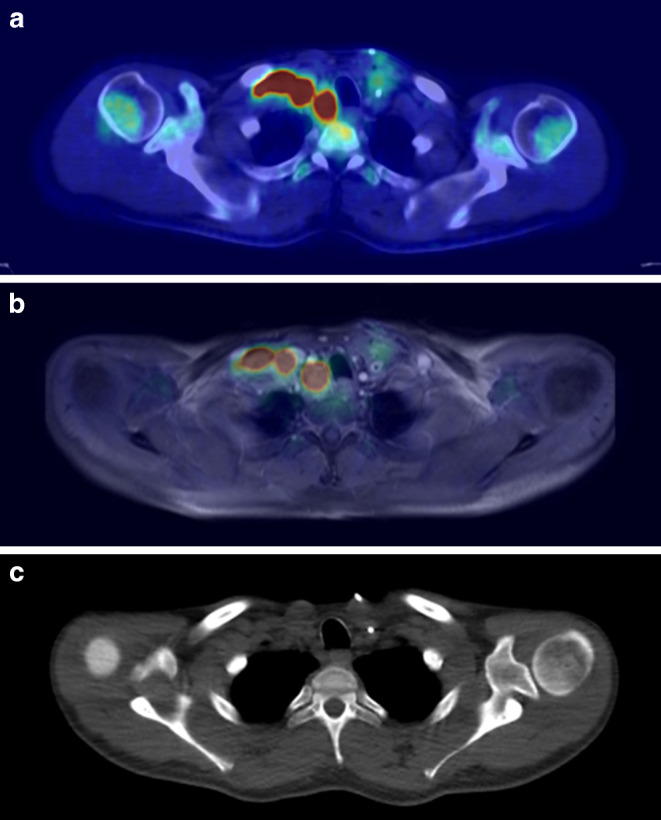
Imaging of patient 4. **a** FDG-PET/CT before chemotherapy. **b** FDG-PET/MRI before chemotherapy. **c** Planning CT after chemotherapy

The sequence of performed scans and therapeutic interventions is depicted in Fig. 1 and exemplarily illustrated for patient 4 in Fig. 2.

**Fig. 3:**
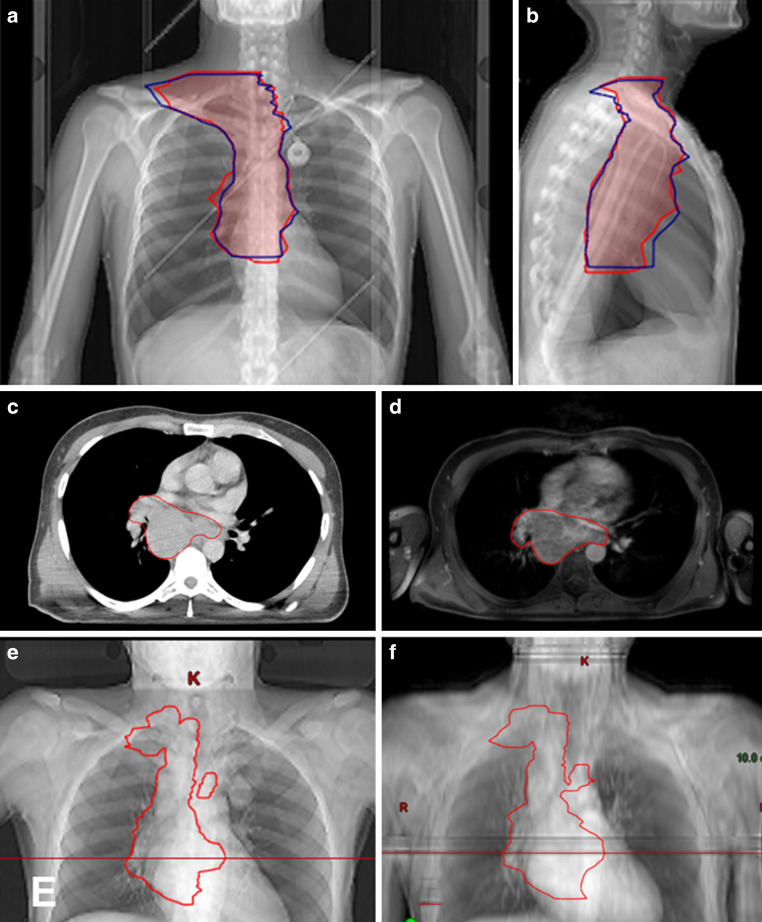
GTV and CTV comparison (patient 4). **a**, **b** Red area: CTV based on PET/CT. Blue outline: CTV based on PET/MRI. **a** frontal view, **b** lateral view. **c**, **d**: Red area: GTV based on PET/CT (**c**) and PET/MRI (**d**). **e**, **f** Corresponding topograms with marking of the section plane

To plan the treatment according to International Lymphoma Radiation Oncology Group (ILROG) guidelines, the initial diagnostic scans were registered to the planning CT to facilitate delineation of the target volumes and render the process more precise [2]. Differing from the cited guidelines, two prechemotherapy gross tumor volumes (GTV) were delineated, one based on PET/CT (GTV-PETCT) and one on PET/MRI (GTV-PETMRI). Accordingly, two clinical target volumes (CTV-PETCT and CTV-PETMRI) were created (Fig. 3).

Qualitative differences between the GTVs regarding the involved lymph node areas were evaluated. The differences were quantified using the Dice similarity coefficient. This coefficient is a measure to assess the concordance between two volumes. It ranges from 0 to 1, with a value of 1 representing perfect overlap and a value of 0 indicating no overlap. The Dice coefficient is defined as [29]

$$\begin{aligned}&\text{DICE coefficient}=&&\mathbf{2}(\text{GTV-PETCT}\cap \text{GTV-PETMRI})\\&&& /(\text{GTV-PETCT}+\text{GTV-PETMRI})\end{aligned}$$

The information from both CTVs was used to create a final CTV. The planning target volume (PTV) was defined by adding a margin of 0.5–1 cm to account for possible setup errors.

The Varian Eclipse software version 15.1 (Varian Medical Systems, Palo Alto, CA, USA) was used to delineate the different volumes and to generate the Dice similarity coefficients.

## Results

### Staging

In all five patients, no differences were observed in the resulting tumor staging between PET/CT and PET/MRI. The patient characteristics are shown in Table 1.

### Radiation therapy planning target volumes

Two patients (1 and 5) received chemotherapy, but RT was not indicated according to the GHSG guidelines due to a more advanced stage of disease diagnosed by PET/CT and PET/MRI. Therefore, no planning CT scan was performed and no further analysis of a possible influence on the CTV regarding PET/MRI or PET/CT was possible. The other three patients received RT in our department. The CTVs contoured in the planning CT based on the different GTVs differed slightly in size (range 0.2–5.4%; Table 1). The Dice coefficients ranged from 0.86 to 0.89 (mean 0.88), indicating a good overlap between GTV-PETCT and GTV-PETMRI.

## Discussion

The use of PET/CT has contributed to a paradigm shift in the treatment of Hodgkin’s lymphoma. It allows precise staging and thus better stratification of treatment regimens and, ultimately, de-escalation of therapy in appropriate cases. The trend towards personalized and optimally stratified treatment should be consistently pursued. Therefore, the burden on patients of diagnostic procedures should be reduced as much as possible; PET/MRI offers a good starting point due to its reduced exposure to ionizing radiation. In our cohort, the PET components from the used PET/CT and PET/MRI devices have comparable sensitivity, and the institutional radioactivity prescription is identical for both. Therefore, the difference in effective dose between PET/CT and PET/MRI is solely attributable to the CT scan. Typical values for CT scanning in Germany are given in a publication by Bos and coworkers [6]. The risk from ionizing radiation is especially high for children and adolescents, so that using PET/MRI instead of PET/CT would be especially beneficial for these patient groups [14]. The use of PET/MRI in lieu of PET/CT, if available and possible, is recommended by the German Commission on Radiological Protection [28]. It may also provide better soft tissue contrast, which is relevant in cases with organ manifestations. To justify clinical implementation of PET/MRI, the diagnostic value of the novel method must be non-inferior and ideally superior to the established method. For our analysis, two critical factors were prioritized: firstly, achieving high accuracy in determining the extent of disease progression (accurate staging) and secondly, carefully positioning the patient to facilitate optimal treatment planning.

### 1. Staging

There are different studies examining staging in pediatric lymphoma patients and adult patients with Hodgkin’s lymphoma. Sher et al. compared the diagnostic performance of PET/MRI and PET/CT in a pediatric cohort of 25 patients. No statistically significant differences were found between either modality regarding detection rates, lesion classification, or Ann Arbor staging. Picardi et al. assessed 60 patients with HL in a prospective setting. Only the bony infiltration detection rates showed a statistically significant difference, but the exposure to radiation was estimated to be fourfold higher with PET/CT than with PET/MRI [23, 24]. Consistent with these findings, our cohort revealed analogous results: PET/CT and PET/MRI lead to similar lymphoma staging in five HL patients. This finding is best explained by the dominant diagnostic value of PET in both scan types. Information from CT and MRI is crucial for anatomical correlation of PET findings. However, in the case of FDG-avid lymphatic diseases, any additional informational gains regarding additional potentially involved lesions appear negligible. It can be concluded that PET/MRI is equivalent to PET/CT in the staging of HL, if readily available.

### 2. Positioning:

From the perspective of the radiation oncologist, apart from staging, optimal reproducibility of the tumor volume in the subsequent radiotherapy planning CT is also required [25].

The staging PET/CT/MRI is registered with the subsequent postchemotherapy radiation planning CT. Correct co-registration of PET/MRI and PET/CT with the planning CT scan facilitates target volume delineation. Performing an initial PET/CT in the planned RT position allows easy identification of initially affected nodes and correlation with the extent of involvement at the time of RT.

Ideally, PET imaging should always be performed in the radiation position. Unfortunately, this is not yet feasible in many clinical situations. Therefore, it frequently falls within the responsibility of the radiation oncologist to delineate an ideal target volume despite potentially suboptimal imaging.

On a standard PET/CT scan, i.e., not set up for radiation therapy, the arms are generally elevated. A study by Bird et al. demonstrated that when relying on a diagnostic PET/CT for contouring, the CTV in the head and neck region needs to be extended by 10 mm cranially and 18 mm caudally, and also more generously contoured in the axial direction when compared to a PET/CT in the radiation position [3].

The PET/MRI registration is less precise due to the different patient positioning inside the MRI scanner. The whole-body coil and curved table result in a fairly compact position. However, PET/MRI has the advantage that the arms are positioned downwards as standard.

In our cohort of optimally positioned PET/CT and PET/MRI scans with lowered arms, contouring of clinical target volumes (CTVs) revealed minor discrepancies in three patients. However, these differences were negligible and likely attributable to intra-observer variability (Dice coefficients of 0.86 to 0.89). Despite this fact, it is difficult to assess the potential impact of PET/CT or PET/MRI on RT planning on a statistical basis.

The radiation oncologist performing the contouring must be aware of the differences between the scans. Moreover, even if these differences did not result in significantly different CTVs in our patients, they could in some cases favor errors.

Two possible disadvantages of PET/MRI have not yet been mentioned. Firstly, the smaller gantry compared to PET/CT means that individual patients, especially those who are very obese, do not fit into the device. Secondly, the examination time can be significantly longer compared to PET/CT. This can be a problem for claustrophobic patients in particular. In nervous patients the examination can quickly be compromised by artifact formation or, in extreme cases, may not be feasible. The PET/CT scanners, on the other hand, usually have significantly larger gantry openings. In addition, the patients are not further restricted by coils. This can lead to significantly better tolerability and ultimately to less artifact formation during imaging in the aforementioned patient groups.

In our cohort, we had no problems with patient size and the small gantry, but in everyday practice this could potentially lead to problems. With regard to the examination time, sensible selection of the MRI sequences is of great importance. Georgi et al. have published a paper in this regard in which recommendations for the selection of sequences are given. Transversal T2w sequences with fat saturation seem to be optimal here and should spare the patient from long examination times. The use of contrast agents showed no advantage and was not recommended [12].

From an economic perspective, it should be noted that PET-MRI is generally significantly more expensive than PET-CT. This is due to the high cost of the device, its maintenance, and the more complex examination technique with correspondingly longer examination times. In addition, PET-MRI devices are often only available in large tumor centers.

## Conclusion

Positron-emission tomography combined with computed tomography is a well-established modality for staging of lymphoma. A PET/CT scan prior to chemotherapy in the radiotherapy position can facilitate delineation of tumor and treatment volumes. However, replacing the CT part with MRI may reduce the patient’s radiation exposure. An additional benefit is that MRI scans are performed with the arms lowered, eliminating the need to modify the acquisition protocol, unlike for PET/CT.

In our albeit small cohort, no significant differences in diagnostic performance or the delineated RT volumes were observed. Thus, PET/MRI is a viable option to ensure adequate staging and subsequent target delineation in patients with HL. Due to the reduction of radiation exposure compared to PET/CT, it may be the preferred option if readily available.

## Data Availability

Please contact Kai Kröger regarding data availability.
